# Analysis, Development, and Scaling-Up of Poly(lactic acid) (PLA) Biocomposites with Hazelnuts Shell Powder (HSP)

**DOI:** 10.3390/polym13234080

**Published:** 2021-11-24

**Authors:** Laura Aliotta, Alessandro Vannozzi, Daniele Bonacchi, Maria-Beatrice Coltelli, Andrea Lazzeri

**Affiliations:** 1 Department of Civil and Industrial Engineering, Pisa University, 56122 Pisa, Italy; alessandrovannozzi91@hotmail.it (A.V.); andrea.lazzeri@unipi.it (A.L.); 2 National Interuniversity Consortium of Material Science and Technology (INSTM), 50121 Florence, Italy; 3 Arianna Fibers s.r.l, 51100 Pistoia, Italy; d.bonacchi@ariannafibers.com

**Keywords:** biocomposites, natural fibers, poly(lactic acid) (PLA), extrusion compounding

## Abstract

In this work, two different typologies of hazelnuts shell powders (HSPs) having different granulometric distributions were melt-compounded into poly(lactic acid) (PLA) matrix. Different HSPs concentration (from 20 up to 40 wt.%) were investigated with the aim to obtain final biocomposites with a high filler quantity, acceptable mechanical properties, and good melt fluidity in order to be processable. For the best composition, the scale-up in a semi-industrial extruder was then explored. Good results were achieved for the scaled-up composites; in fact, thanks to the extruder venting system, the residual moisture is efficiently removed, guaranteeing to the final composites improved mechanical and melt fluidity properties, when compared to the lab-scaled composites. Analytical models were also adopted to predict the trend of mechanical properties (in particular, tensile strength), also considering the effect of HSPs sizes and the role of the interfacial adhesion between the fillers and the matrix.

## 1. Introduction

Due to their complex end-of life management, petroleum-based plastics have caused a serious environmental problem, mainly related to their disposal. It was observed that from 1950 to 2015, less than 10% of the total plastic produced amount was recycled [1]. A possible solution to the waste management problem caused by non-degradable plastics can be obtained by replacing these materials with biodegradable polymers obtained from renewable resources compounded with agro-food waste. In this context, biobased and biodegradable polymers are an interesting solution to preserve petroleum resources and to decrease CO_2_ emissions [2].

Agro-industry generates large biomass amounts that are not sufficiently and adequately exploited. For example, in the European Union alone, about 700 million tons of agriculture waste is annually produced [3]. The use of plant waste materials as raw materials in the production of biocomposites materials represents an exceptional opportunity for sustainable technological development. In fact, fruit shells and other agricultural waste are potentially important sources for the production of sustainable and competitive biocomposites. These plant by-products are produced in high quantities and crop wastes are rich in different nutritional components that can be valorized. Recently, the utilization of by-products has been increased by food and pharmaceutical manufacturers to produce valuable compounds from such inexpensive resources. In particular, nuts are one of the most important agricultural products due to their different uses within the food industry [4]. 

Walnut and hazelnut shells have great potential due to their large scale production; considering that about 67% of the total product weight consists of the shell, 646,818 tons of walnut shells, and 353,807 tons of hazelnut shells are produced each year [5]. After the separation of the kernel from the external parts of the fruit, large quantities of peel and shell are generated. These materials are the main part (over 60%) of the nut fruit and are discarded or burned as fuel without any useful application. Unfortunately, this waste material is typically burned directly in situ for heating purposes, while it could potentially be used for the production of both high added-value chemicals and biocomposites. Hazelnut shells are cost-effective byproducts [6] and their exploitation represents a stimulating challenge [7]. To better exploit their potentialities, it is necessary to find other better uses for hazelnut shells [8,9]. Hazelnut shells’ composition is very similar to that of other wood-based biomass because cellulose, hemicellulose, and lignin are the main components. Shell grinding allows to produce hazelnut shell powder (HSP) of different sizes and morphologies. HSPs consist of lignin (40–50% by weight), cellulose (25–28%), and hemicellulose (22–30%), but they also contain a fraction of polyphenols (flavonoids and tannins), which can be recovered by hydroalcoholic extraction [10,11,12]. The shell extracts can be used as natural antioxidants in polymeric matrices as they can act as thermal and photo oxidative stabilizers for different types of polymers, including biopolymers like poly(lactic) acid (PLA) [13,14]. Moreover, the HSP addition enables light biocomposites to be obtained that, in some cases, possess improved mechanical and thermal properties and have enhanced biodegradability, when compared to the pure matrices [15,16,17,18,19]. Furthermore, the incorporation of HSPs into a biopolymeric matrix contributes to reducing the overall biocomposite cost [20]. However, some drawbacks must be mentioned in using agricultural waste for the production of lignocellulosic composites: unstable fiber availability over the year, absence of industrialized processing, and the need for big storage facilities and different necessary pre-treatments [21,22,23,24]. For this purpose, in order to take a step forward, the extrusion and injection molding processes considering the biocomposites scaling-up ability were investigated in this paper.

The polymeric matrix chosen for this study was poly(lactic acid) (PLA). In fact, among the biopolymeric matrices commercially available in the market, poly(lactic) acid (PLA) is one of the most attractive and its use in the production of green composites is gaining great importance [25]. PLA can be considered the front runner of the bioplastic market with an annual consumption of about 140,000 tons [26]. What has pushed up the increasing PLA demand are its excellent starting mechanical properties (≈3 GPa of Young’s modulus, ≈60 MPa of tensile strength, ≈3% of elongation at break and an impact strength close to 2.5 kJ/m^2^) that are comparable to those of polystyrene (PS) [27]. 

Song et al. investigated the addition of walnut shell powder into PLA; they noticed during the biocomposites processing that an increase in the melt fluidity was correlated to the fiber powder addition [28]. This melt fluidity increment can lead to problems during the processing, making impossible or very difficult, for example, the extrusion compounding, the injection molding, the casting extrusion, etc. The evaluation of the fiber/matrix adhesion plays an important role and must be considered. From the processing point of view, fiber-matrix adhesion improvement can be done by chemical fiber pre-treatments or in-situ reactive blending. The last option is very interesting for the scaling-up point of view and involves the use, during the extrusion compounding, of coupling agents that are able to modify the polarity and surface tension of the fibers, enhancing the fiber-matrix adhesion [29,30]. The main coupling agents added to improve the fiber-matrix adhesion are maleic anhydride (MA), silane, isocyanate, and peroxide [29,31,32]. Commercial chain extender represents another way to improve the fiber-matrix adhesion, thanks to their easy processability during the extrusion compounding; however, they are not bio based and not biodegradable and even if they are introduced in very few amounts, they compromise the totally full bio-based origin of the final biocomposites.

The addition of HSPs into a PLA matrix must be deeply investigated and little work has been done regarding the scaling-up of these biocomposites into semi-industrial extrusion compounding process. For this reason, in this work, firstly the effect of the addition of different amounts (from 20 up to 40 wt.%) of two HSPs with different values of granulometry was investigated. The effect on melt fluidity, and thermal and mechanical properties was investigated on a lab-scale. Analytical models were also adopted to evaluate the powder size effect and adhesion between HSPs and PLA matrix. Then, the best selected compositions were extruded into a semi-industrial twin screw extruder, evaluating scale-up feasibility, focusing on the change of melt fluidity and mechanical properties of the scaled-up composites. 

## 2. Materials and Methods

### 2.1. Materials

The materials used in this work are:

- PLA3251D from Natureworks is a PLA designed for injection-molding applications. This polymer grade is very stable in the molten state and can be processed on conventional injection molding equipment [density: 1.24 g/cm^3^; MFR (210 °C, 2.16 kg): 80 g/10 min].
- Two different KERN hazelnut shell powders (HSPs) with different granulometry were provided by Arianna Fibers. Empty hazelnut shells were grounded by an impact mill. HSP with coarser grain size are named H0210, while those with finer grain size are named HM200 [ρ = 0.954 to 1.08 g/cm^3^ with HR 5 to 30%].

### 2.2. Hazelnut Shell Powders (HSPs) Characterization

In order to quantify the humidity present in the HSPs, about 0.5 g of HSP for each sample were put in a Petri dish (previously weighed) and they were weighed before and after the drying process in a ventilated oven at 60 °C for 16 h. For each fiber typology, at least 3 measurements were carried out. 

To investigate the possible degradation of the fillers during the extrusion compounding and to evaluate differences in chemical compositions between H0210 and HM200 HSPs, thermogravimetric (TGA) and FT-IR analysis were carried out.

TGA was performed on a TA Q-500 instrument (TA Instruments, Waters LLC, New Castle, DE, USA). Few milligrams were heated at 10 °C/min from room temperature up to 700 °C at 10 °C/min in nitrogen atmosphere.

FT-IR analysis was carried out on a Nicolet T380 FT-IR (Thermo Scientific, Madison, WI, USA) spectrometer equipped with an ATR Smart iTX accessory. Infrared spectrum of HSP was recorded in the 550–4000 cm^−1^ range, collecting 256 scans at 4 cm^−1^ resolutions.

The powders morphology was investigated by scanning electron microscopy (SEM) analysis using a FEI Quanta 450 FEG (Thermo Fisher Scientific, Waltham, MA, USA). The samples were prior sputtered with platinum to enhance their conductivity and generate the images, thanks to the secondary electrons. For each fiber typology, different images were acquired in order to obtain the filler distributions. The HSPs distributions were obtained, according to literature [33,34], measuring the dimensions of at least 200 filler particles by using Image-J software.

### 2.3. Lab-Scale and Semi-Industrial Scale-Up Extrusion Compounding and Injection Molding

PLA based composites containing different HSP amounts (from 20 up to 40 wt.%) were extruded at laboratory scale with a Haake Minilab II (HAAKE, Vreden, Germany) twin-screw mini-compounder. Before the extrusion, all materials were dried in a Piovan DP 604–615 dryers (Piova S.p.A., Verona, Italy) at 60 °C for 16 h. The extrusion temperature was set at 190 °C with a mixing residence time inside the extrusion chamber of 40 s and a screw speed of 60 rpm. The strand coming out from the mini extruder was then cooled and pelletized to obtain granules. The composites name and their compositions are reported in Table 1.

**Figure 1:**
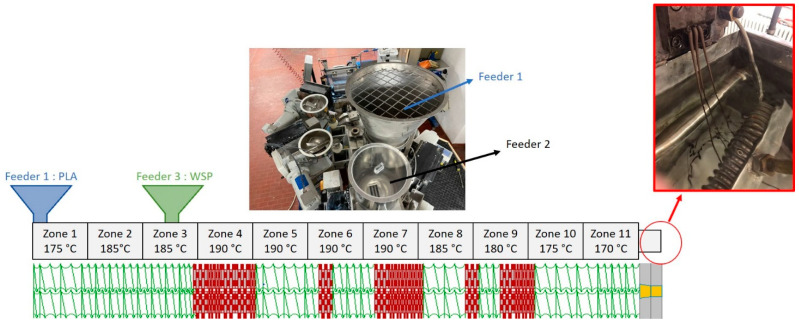
Schematization of the semi-industrial Comac twin screw extruder. In the figure are highlighted the feeder position, the screw configuration and the profile temperature along the 11 extruder zones.

To the best composition of both HSP typologies, the extrusion compounding was scale-upped on a semi-industrial Comac EBC 25HT (L/D = 44) (Comac, Cerro Maggiore, Italy), twin screw extruder. Also, in this case, the materials were dried following the same procedure adopted for the mini-compounding. PLA pellets were introduced into the main feeder while HSPs were fed with a specific lateral feeder that allows, once that the weight concentration was set, a constant feeding rate during the extrusion. A schematization of the extrusion feeder configurations, as well as the temperature profile adopted in the 11 extruder zones, is reported in Figure 1.

The strands coming out from the extruder were cooled in a water bath and then pelletized by an automatic cutter. After the extrusion (both in lab-scale and in scale-up process), the pellets (dried in the before mentioned Piovan dryer at 60 °C for 16 h) were injection molded with a Megatech H10/18 injection molding machine (TECNICA DUEBI s.r.l., Fabriano, Italy) to obtain ISO 527-1A dog-bone specimens (width 10 mm, thickness 4 mm, useful length 80 mm) and ISO 179 Charpy impact specimens (width 10 mm, thickness 4 mm, length 80 mm). The injection molding was carried out in order to minimize any change in the processing parameters (reported in Table 2) for a better understanding of melt viscosity variation induced by the addition of different quantities and different HSP typology (H0210 and HM200). Consequently, the temperature profile, the mold temperature, the injection time, and the cooling time were fixed and only the injection pressure was modified when necessary. 

### 2.4. Melt Flow Rate (MFR)

In order to evaluate the melt fluidity variation caused by the addition of HSP, the melt flow rate (MFR) were measured on the biocomposites pellets by a CEAST Melt Flow Tester M20 (Instron, Canton, MA, USA) equipped with an encoder. The standard ISO1133D method was used: the sample was preheated without any weight for 30 s at 190 °C and then a weight of 2.16 kg was applied. The molten material quantity that flows for 30 s was then weighted and the MFR calculated. At least three measurements for each composition were carried out and the mean MFR value reported. Before the test, the materials were kept in a ventilated oven at 60 °C to avoid the pellets water uptake.

### 2.5. Mechanical and Thermal Characterization

Tensile tests were carried out on the ISO 527-1A extrusion molded specimen using an MTS Criterion model 43 (MTS Systems Corporation, Eden Prairie, MN, USA) universal testing machine. The MTS was equipped with a 10 kN load cell and the crosshead speed was set at 10 mm/min. Tensile tests were performed, at room temperature, after 3 days after the sample injection molding and during this time, the sample were stored in a dry keep at 25 °C and 50% of relative humidity. At least six specimens for each composition were tested.

Charpy impact tests were carried on the injection molded specimen pre-notched with a V-notch of 2 mm. A CEAST 9050 machine (INSTRON, Canton, MA, USA) was used and at least six specimens, at room temperature, were tested. The impact tests, also in this case, were carried out after 3 days of the injection molding keeping the samples in a controlled atmosphere. 

The main biocomposites; thermal properties were calculated by differential scanning calorimetry (DSC) using a Q200-TA DSC (TA Instruments, New Castle, DE, USA) equipped with an RSC 90 cooling system. Nitrogen was used as purge gas set at 50 mL/min. Few milligrams (about 12 mg) were cut from the injection molded samples and the heating program was set in order to consider the thermal history of the samples and thus considering the injection molding history. In this way it was possible to calculate the crystallinity reached by the samples after the injection molding process. The thermal program was: heating at 10 °C/min from room temperature up to 200 °C, the final temperature was kept for 1 min. The melting and crystallization temperatures were calculated in correspondence of the maximum and minimum of the melting peak and cold crystallization peak, respectively. As far as the melting and cold crystallization enthalpies were concerned, they were calculated integrating the peak areas of the melting and crystallization peaks, respectively. The PLA crystallinity percentage of PLA was calculated according to the following equation (Equation (1)) [27]:

(1) $$X_{cc}=\frac{\Delta H_{m}-\Delta H_{cc}}{\Delta {H{}^{\circ}}_{m}\cdot wt.{\%}_{\mathrm{PLA}}}$$

where, Δ*H_m_* and Δ*H_cc_* are the melting and cold crystallization PLA enthalpies of PLA, Δ*H°_m_* is the theoretical melting heat of 100% crystalline PLA (taken equal to 93 J/g [35]). 

### 2.6. Composite Morphology Investigation

The composites morphology was investigated on the fractured cross-sections of the Charpy samples prior the sputtering with platinum. A FEI Quanta 450 FEG scanning electron microscope (SEM) equipped with a Large Field Detector for low kV imaging simultaneous secondary electron (SE) was used.

## 3. Theoretical Analysis 

During the lab scale investigation, different analytical models were applied on the HSP/PLA based composites to estimate the fiber/matrix adhesion and to predict the tensile strength trend as a function of the HSPs volumetric content. The addition of rigid particles into a polymeric matrix can affect the strength in two ways. The tensile strength prediction of particulate filled composites is not easy because it is affected by different parameters, such as interface adhesion, stress concentration, and defect size/spatial fillers distribution [36]. 

For particulate fillers and for fibers with low aspect ratio, the prediction of the tensile strength can be expressed quantitatively by the following equation, proposed by Pukánszky [37]:

(2) $$\sigma_{c}=\sigma_{m}\left[\frac{1-V_{f}}{1+2.5V_{f}}\right]\exp\left(BV_{f}\right)$$

where, *σ_c_* and *σ_m_* are the stress at break of the composite and matrix, respectively, while *V_f_* is the volume fiber fraction. The term in square bracket is correlated to a decrement of the tensile strength of the composite caused by the fillers addition that reduce the load-bearing cross-section of the composite. The parameter *B* is an interaction parameter that takes into account the efficiency of the stress transmission between the matrix and the filler and can be indirectly correlated to the filler/matrix adhesion [38]. Simplifying Equation (2), a linear correlation can be obtained (Equation (3)) in which the B parameter is found as the slope of the natural logarithm of reduced strength (*σ_red_*) against the volume filler fraction.

(3) $$ln\sigma_{red}=ln\frac{\sigma_{c}\left(1-V_{f}\right)}{\sigma_{m}\left(1+2.5V_{f}\right)}=BV_{f}\;\;$$

For particulate fillers, in the case that the stress cannot be transferred from the matrix to the filler and the final composite tensile strength is determined from the effective sectional area of the load-bearing matrix, the tensile strength of the composites lies between an upper and lower bound [36]. Based on the hypothesis that poor adhesion exists between the filler and the polymer and the load is sustained completely by the polymer matrix, the following equation (Equation (4)) formulated by Nicolais and Nicodemo [39] gives the lower-bound strength of the composite.

(4) $$\sigma_{c}=\sigma_{m}\left(1-1.21V_{f}^{\frac{2}{3}}\right)$$

The upper bound is immediately obtained as follows (Equation (5)):

(5) $$\sigma_{c}=\sigma_{m}\left(1-V_{f}\right)$$

Equation (5) generally has been considered as an ideal unattainable upper bound since, in addition to a matrix area reduction, critical effects are also induced by the filler particles in the system, with a further decrease of the composite strength. 

## 4. Results 

### 4.1. HSPs Characterization Results

The results of the HSP drying tests showed that H0210 had a humidity loss of about 9.05%, while for HM200 it was 7.64%. HSPs having lower particle size release less moisture after the drying. 

**Figure 2:**
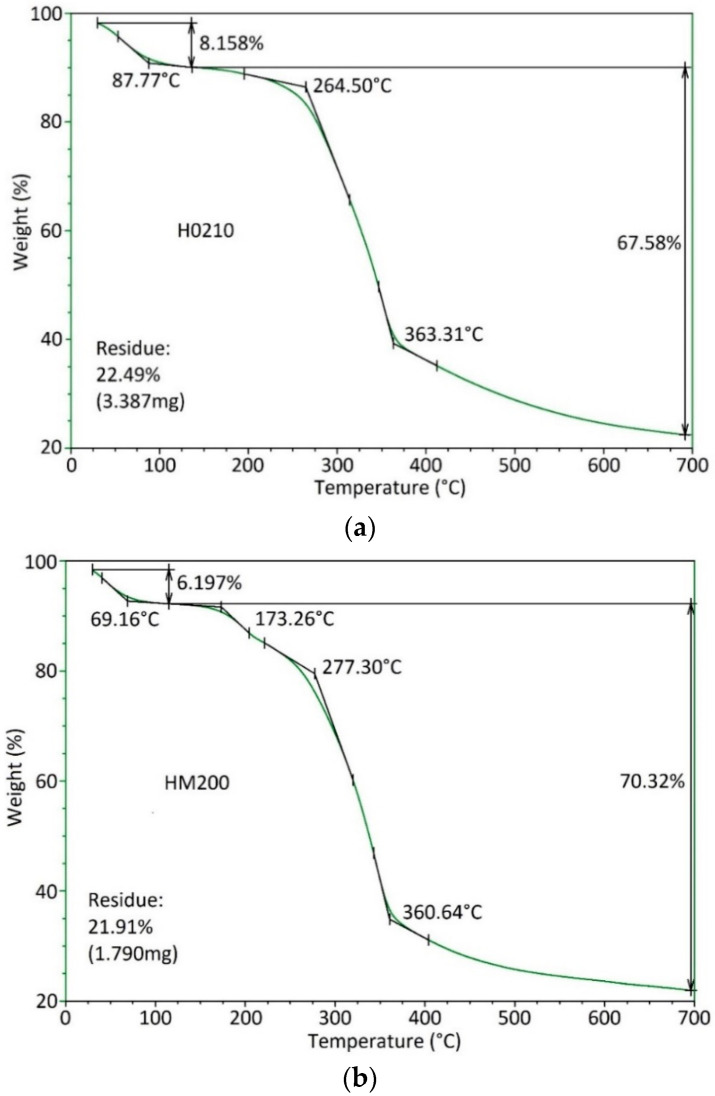

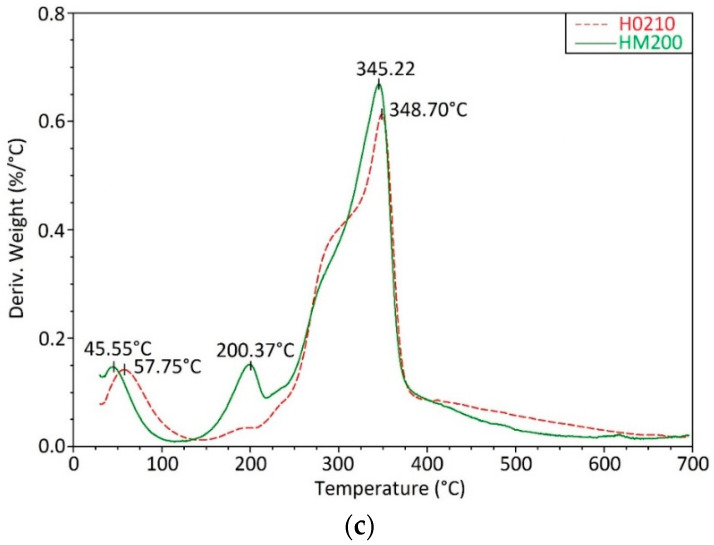
TGA curves of (**a**) H0210 and (**b**) HM200 hazelnut shell powder. Figure (**c**) illustrates the overlay of the TGA curves derivative.

From the TGA results reported in Figure 2, it can be observed that, for both HSP typologies, the thermograms are characterized by a first mass drop (completed below 100 °C) that is correlated to the humidity loss of the HSPs. The moisture loss is greater for the H0210 sample, indicating that the HSP having higher particle size dimension releases easily the up taken water. The second mass drop for H0210 corresponds to the thermal degradation of the material and presents a similar magnitude for the two HSP typologies. The final residue is also similar in magnitude for both samples. The residue includes both inorganic compounds and carbon, normally generated when thermal degradation occurs in a nitrogen atmosphere. The superimposed derivatives of the curve show the inflection point (where the mass loss occurs) as a maximum. The main maximum peak is about the same for the two samples; however, HM200 shows an additive peak at around 198 °C probably indicating a major quantity of water highly linked on the surface or substances with lower thermal resistance. 

**Figure 3:**
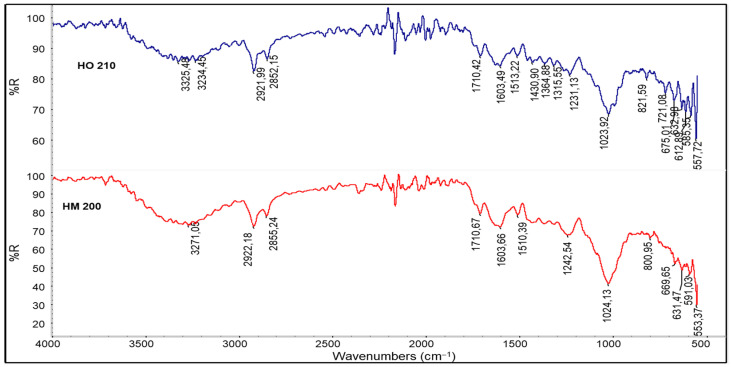
ATR spectra of H0210 and HM200 hazelnut shell powders.

Hazelnut shells are composed of cellulose, hemicellulose, and lignin. However, there is a significant amount of low molecular weight compounds. In literature [11], it was observed that hazelnut shell contains about 10.6% of low molecular weight extractable substances, about 30.1% of lignin and about 49.7% of polysaccharides (cellulose and hemicellulose). From the ATR spectra reported in Figure 3, for H0210 a wide band at around 3327 cm^−1^ was observed, attributed to the surface hydroxyl groups (-OH) mainly related to the presence of water as well as alcoholic, phenolic groups but also amino acids and carboxylic derivatives.

The peak at 2920 cm^−1^ is assigned to the asymmetric stretching band C-H; also, that at 2850 cm^−1^ is related to the symmetrical stretching of the same bonds. These groups are also present in the structure of lignin [40]. The peak associated with the stretching of C=O (carbonyl compounds) is located at 1708 cm^−1^, but a shoulder is noted at 1743 cm^−1^. While the main peak is attributable to carboxylic acids, the second is attributable to the presence of ester groups. The presence of unsaturations and C=C bonds that occurred in the widened bands between 1606–1640 cm^−1^ is attributable to alkenes, aromatic groups, but also amide groups (C=O stretching); while the peaks at 1400 and 1240 cm−^1^ may be due to C−O, C−H or C−C elongation vibrations. The peak observed at 1024 cm^−1^ is due to C–O, present in the ethereal, alcoholic, and carboxylic groups. The band of the C–O group is more intense than that of the C=O group, and this shows that the polysaccharide component is certainly dominated in the sample. The peak at 588 cm^−1^ is due to the folding vibration in the aromatic compounds typical of lignin, highlighting their presence.

The spectrum of HM200 was acquired in a similar way to that of H0210, but the signals are more intense. This is attributable to the lower particle size of the powder, which allows better adhesion of the sample to the crystal. The observed bands are completely similar to those of the H0210 sample, suggesting that the only difference between H0210 and HM200 is in the particle size.

**Figure 4:**
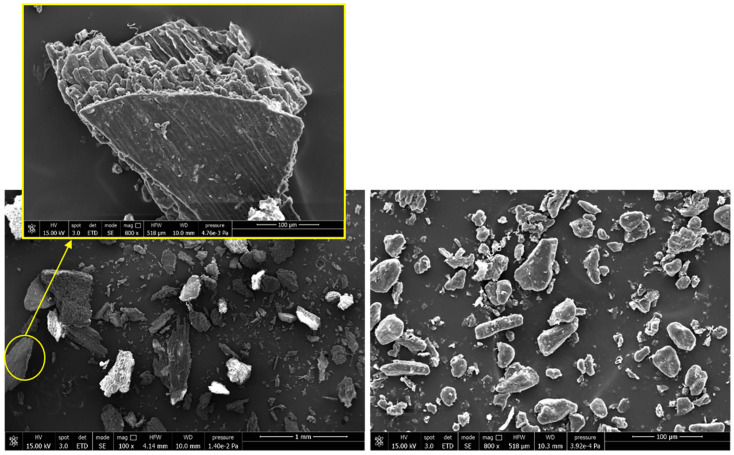
SEM images of H0210 (**left side**) and HM200 (**right side**).

The SEM micrographs (Figure 4) show, especially for H0210, the presence of irregularly shaped particles having a rough surface attributed to the external part of the hazelnut shell, which has a different morphology, depending on the filler layers. For smaller-sized samples, the amount of rough surface particles is reduced.

**Figure 5:**
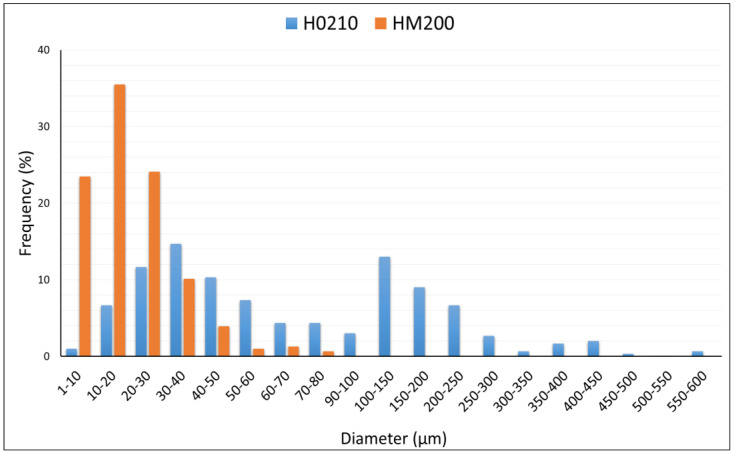
Diameter distributions for H0210 and HM200 HSP.

Also cavities and reliefs are visible that correspond to the cell walls and lumens. In any case, for both HSPs, a greater variability in the filler shape and size can be observed. The morphological results are consistent to what can be found in literature and despite the different surface roughness, both HSPs can be considered as a typical particle-shaped fillers [41,42]. The “elliptical approach” was adopted to determine the diameter distribution; according to this model, the major axis of the ellipse corresponds to the length of the filler while the minor axis corresponds to the width. With this method, the length and aspect ratio are overestimated by about 10% for the fiber-shaped filler while this overestimation is practically negligible for the particulate filler [34]. Since from the SEM images the greater quantity of HSPs tends to be particle, with little presence of elongated fibers, it was preferred to adopt this elliptical model. In Figure 5 the diameter distribution curves are shown that confirm the great differences in diameters dimension between H0210 and HM200. In particular, an average diameter of 206.7 μm and 25.8 μm was obtained for H0210 and HM200, respectively.

### 4.2. Lab-Scaled Composites Results

The results of mechanical tests and MFR are summarized in Table 3. From the point of view of tensile tests, the powders’ addition makes the material more brittle with a decrement of both stress and elongation at break. The HSPs addition, on the other hand, significantly increases the elastic modulus compared to pure PLA. This is a common trend [43] and it is due to the introduction of fillers having higher elastic modulus than pure matrix. In general, a decrement of the mechanical properties increasing the HSPs amount can be observed; however, for H0210, the tensile decrement is less marked than HM200 and the impact resistance is not worsened with respect to pure PLA.

**Figure 6:**
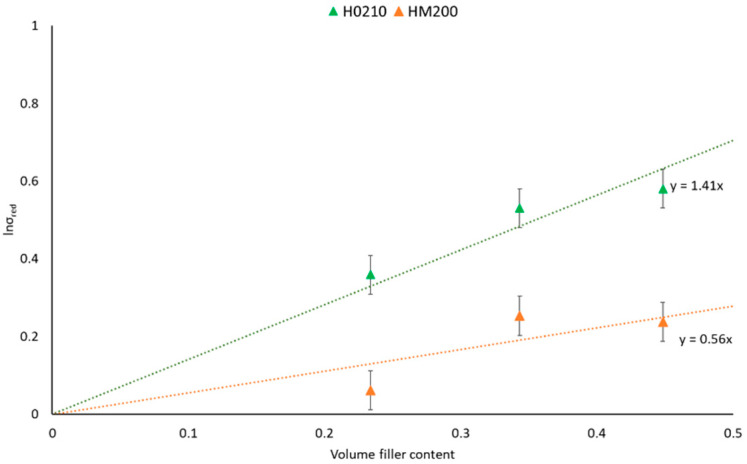
Pukanszky’s plot for PLA-HSPs composites.

The better mechanical response achieved with H0210 can be attributed to several factors. First of all, H0210 possesses a greater diameter distribution that represents a more efficient obstacle towards the crack that advances during the Charpy test, compared to HM200 with a finer diameter distribution [36]. Furthermore, residual moisture content must also be considered because it also affects the final mechanical response. H0210, under the same drying conditions, lost a greater amount of moisture that could potentially degrade the PLA (it must be considered that in this first lab-scale step, no venting for the humidity stripping is present in the mini-extruder). Finally, the filler/matrix adhesion must also be considered. It is well known from the literature that natural fibers have poor adhesion with PLA [44]. However, comparing HM200 and H0210, the fillers with higher grain sizes will have greater adhesion (the stress able to cause the fiber detachment is in fact a function of various parameters, including the aspect ratio) [45]. The different adhesion is also confirmed by the B parameter obtained as the slope of the Pukanszky’s plot (Figure 6). A decrement of the B value (from 1.41 for H0210 to 0.56 for HM200) can be observed, indicating a worsening of the matrix/filler adhesion.

**Figure 7:**
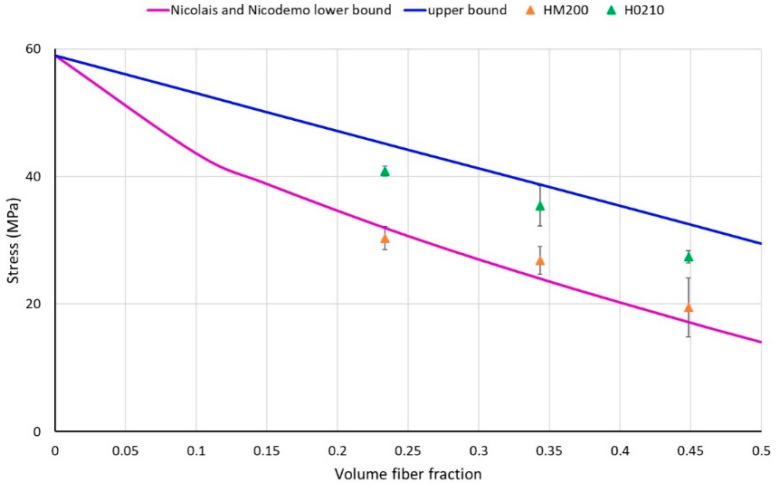
Comparison between the experimental composite strength and the values predicted according to the upper and lower bound equations.

Observing in addition the experimental values of composites tensile strength (Figure 7), a different interaction between H0210 and HM200 with the PLA matrix can be observed. The experimental data in fact, lies between the upper and lower bound; however, HM200 are much closer to the Nicolais and Nicodemo lower-bound equation indicating a weaker adhesion respect to H0210 that are closer to the upper bound. The particles with smaller size have a great tendency to agglomerate, causing greater weakening of the matrix.

**Figure 8:**
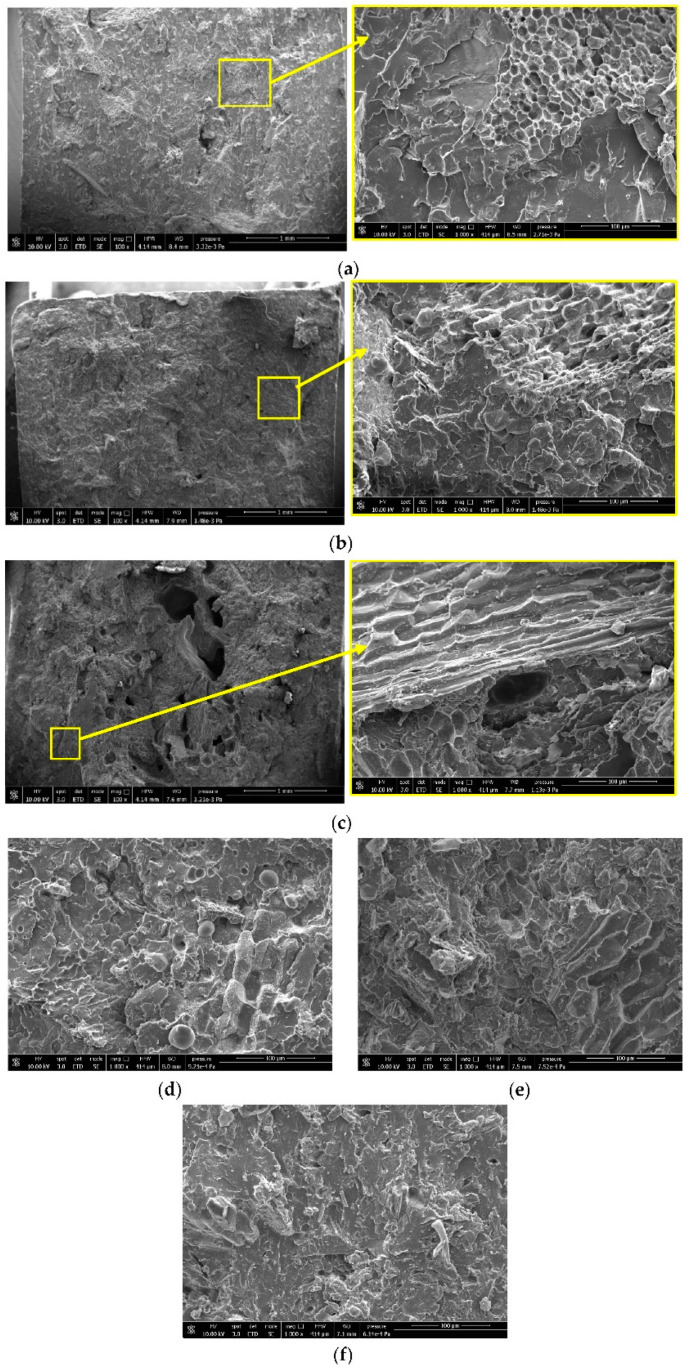
SEM micrographs of the fractured Charpy surface of: (**a**) PLA_20_H0210, (**b**) PLA_30_H0210, (**c**) PLA_40_H0210, (**d**) PLA_20_HM200, (**e**) PLA_30_HM200, (**f**) PLA_40_HM200.

The SEM images reported in Figure 8 confirm the prediction of the analytical models and of the mechanical results obtained. A better adhesion is registered for H0210, respect to HM200. In particular, for H0210, it can be observed that at 20 wt.% (Figure 8a), the fillers are fairly well distributed, and few agglomerations can be observed with 30 wt.% of HSP (Figure 8b). At 40 wt.% however, a greater agglomeration tendency, due to the greater HSPs amount introduced, is registered. The agglomerates are also less adherent to the PLA matrix and in Figure 8c, holes due to the detachment of these agglomerates are clearly visible; the presence of agglomerates is also responsible for the marked drop down of the mechanical properties recorded for the PLA_40_H0210 composite. HM200 show worse adhesion and already at 20 wt.%, voids can be observed due to HSPs’ detachment (Figure 8d). However, for all the compositions (Figure 8d–f), a HSPs detachment can be recorded, which is very marked compared to H0210, confirming the results of mechanical tests and analytical models adopted.

**Figure 9:**
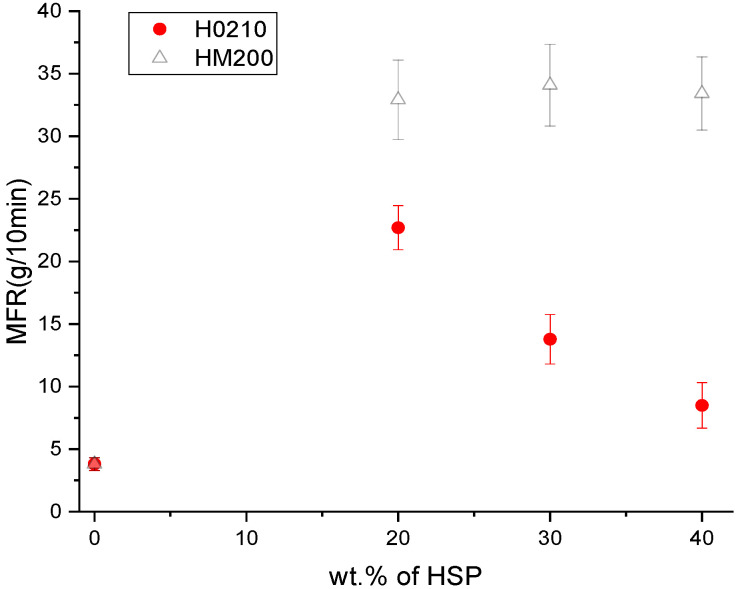
Trend of Melt Flow Rate (MFR) as a function of HSP content.

Regarding the MFR values, it can be observed that the viscosity increased (so the fluidity decreased) on adding the HSPs (Figure 9). However, this occurred only for H0210. For HM200 the MFR values are higher if compared to those obtained with H0210, but no trend with the HSPs amount was detected. These results are in agreement with those reported by Song et al. [28] and with the injection pressure, reported in Table 2, where the injection pressure was increased with the H0210 content while it was decreased by increasing the HM200 content. These MFR trends can be attributable to the probable partial PLA hydrolysis caused by the filler moisture that is greater for HM200 due to its larger surface area and thus humidity content. However, as reported in literature, the general decrement of stress at break is mainly correlated to the poor interfacial adhesion of lignocellulosic fiber with the biopolymeric matrix [45,46].

From a thermal point of view, a decrement of both melting temperature and glass transition temperature caused by the addition of HSPs can be observed (from Table 4); this decrement seems to be correlated to the HSP content. However, the HSP typologies also affect the melting temperature and glass transition temperature differently with a decrement that is more marked with HM200. This behavior can be correlated to the different granulometry between H0210 and HM200. HM200 have a higher surface area than H0210 and it adsorbs more moisture that can lead to decrease in the average molecular weight (resulting in a decrement of the glass transition and melting temperatures). The HSPs’ addition increases the crystallinity of the PLA, causing a shift of the cold crystallization temperature towards lower temperatures. HSP seems to act as a nucleating agent, providing heterogeneous nucleation sites similar to other systems filled with natural fibers [27,47,48]. In particular, as the HM200 are finer and more homogeneous with a tighter diameter distribution curve, they are more effective in crystallizing the PLA, when compared to their H0210 counterparts.

### 4.3. Scaled-Up Composites Results

From the lab-scale data, 30 wt.% seems the most promising HSPs amount granting both a high fiber content and acceptable mechanical properties. The results of the scaled-up composites are summarized in Table 5.

All scaled-up formulations show a lower MFR, respect to their corresponding lab-scale formulations. This MFR decrement is also reflected in the injection pressure increment during the injection molding process (Table 2). The marked viscosity decrement observed during the lab-scale step is limited, thanks to the coupling of the low extruder residence time and the presence of the venting system connected to a vacuum pump that guarantees the humidity stripping during the melt extrusion, avoiding or limiting any eventual PLA degradation [49,50]. The mechanical results are noteworthy. In fact, it can be observed that the scaled-up composites show an increment of elastic modulus and tensile strength. In particular, the reached tensile stress is very similar, confirming the efficiency of the venting system in removing the fillers humidity.

The thermal properties (Table 6) of the scaled-up composite remains almost unchanged, confirming the nucleation effect of HSPs.

## 5. Discussion

**Figure 10:**
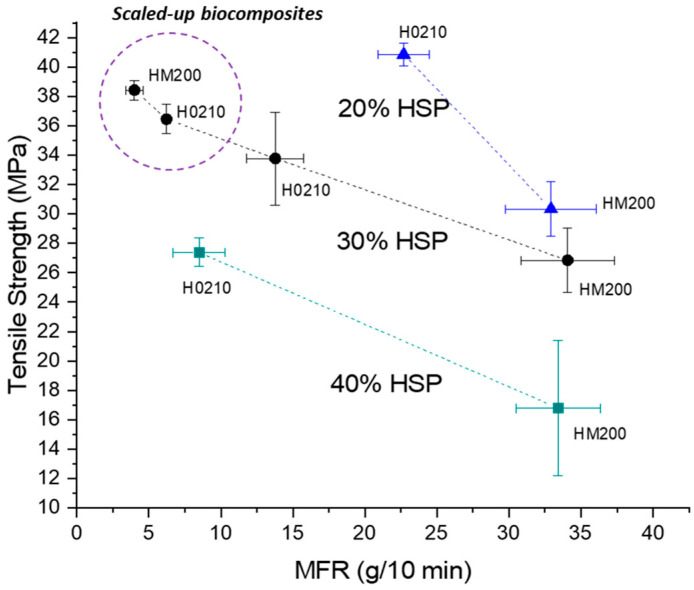
Tensile strength as a function of MFR for biocomposites containing 20, 30, and 40% of HSP.

In attempt to better correlate the obtained results with the mechanical properties, it was noticed that in general the tensile strength of the prepared biocomposites decreased by increasing the melt fluidity, as shown in Figure 10, where the data related to composites containing 20, 30, and 40% of HSP are reported. Interestingly, for the 30% HSP biocomposites, the data obtained for the scaled-up samples follow a similar trend, but as yet observed, the tensile strength is higher and the MFR is lower. Moreover, finer HSP (HM200) results in the highest value of tensile strength and lowest value of MFR. Hence, by avoiding the chain scission of PLA thanks to the optimized processing conditions, the fluidity is greatly decreased.

**Figure 11:**
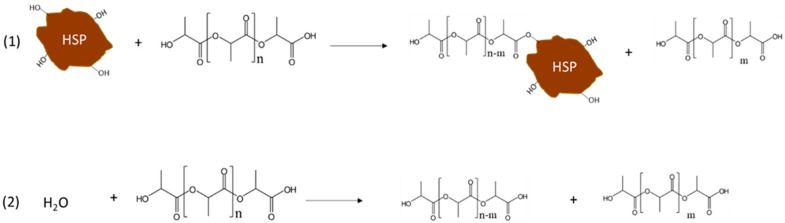
Main reactions occurring during the preparation of PLA/HSP biocomposites; reaction 1 involves an HSP solid particle.

The production of the biocomposites including PLA and HSP resulted in strong interactions or reactions (Figure 11, reaction 1) between the polymer matrix and the functional groups on the HSP surface. Hydroxyl groups, belonging to cellulose and hemicellulose, that represent the major component of HSP, were mainly considered.

Reaction 1’s occurrence depends on the surficial area of HSP and can induce an increase in tensile strength, thanks to the improved matrix-filler adhesion. On the other hand, reaction 2 (Figure 11, reaction 2) is PLA hydrolysis due to humidity, occurring more in the composites containing the finer HSP. In a lab-scale extruder configuration, reaction 2 affects properties more than reaction 1 because of the higher residence time and absence of devolatilization. Thus, as demonstrated by the study of B parameter obtained as the slope of the Pukanszky’s plot (Figure 6), the dispersion in the matrix of the HSP with the lowest dimension was less efficient. On the contrary, when the preparation is scaled-up, reaction 1 as well as the fibre-matrix interaction are more significant. In good agreement, the finer HSP, with the highest surface area, resulted in the highest tensile strength and highest melt viscosity.

## 6. Conclusions

In this study the possibility to process successfully, at the semi-industrial scale, PLA-based composites containing hazelnut shell powder (HSP) was investigated. A first lab-scale production was carried out in order to individuate the best HSPs amount for the subsequent scaling-up step. Two different HSPs typologies of different sizes were added from 20 up to 40 wt.%. The thermal, mechanical, and melt fluidity analysis showed poor stress transfer, which led to a decrement in tensile strength. The fillers seem to act as nucleating sites for PLA that increased its crystallinity; however, a marked decrement of the melt viscosity was recorded, especially for fillers small in size due to their major water uptake. The composition including 30 wt.% of HSP was selected for the successive scale-up in a semi-industrial extruder. Interesting results were obtained considering the scaled-up composites, as their melt fluidity was decreased thanks to the presence of the venting system in the extruder that efficiently removed the residual humidity. The scaled-up composites showed improved mechanical properties, respect to the lab-scaled composites, demonstrating that these composites are effectively processable and can be easily scaled-up The prepared biocomposites showed the possibility of achieving an optimized balance between improvement of mechanical properties and the valorization of a significantly high HSP content.

In future work, a further step towards more efficient exploitation of HSPs should concern their functionalization. The HSPs’ superficial modification, coupled with the optimization of the extrusion process parameters, would allow to obtain biocomposites with further improved mechanical properties.

## Figures and Tables

**Table 1:** Blends name and compositions.

Blend Name	PLA wt.%	HSP wt.%
PLA	100	0
PLA_20_H0210	80	20
PLA_30_H0210	70	30
PLA_40_H0210	60	40
PLA_20_HM200	80	20
PLA_30_HM200	70	30
PLA_40_HM200	60	40
PLA *	100	0
PLA_30_H0210 *	70	30
PLA_30_HM200 *	70	30

* Blends extruded with a semi-industrial COMAC twin-screw extruder (up-scaled).

**Table 2:** Injection-molding parameters.

Blend Name	Temperature Profile (°C)	Mold Temperature (°C)	Injection Time and Cooling Time (sec)	Injection Pressure (bar)
PLA	185–190–190	60	5	120
PLA_20_H0210				90
PLA_30_H0210				90
PLA_40_H0210				95
PLA_20_HM200				70
PLA_30_HM200				70
PLA_40_HM200				70
PLA *				120
PLA_30_H0210 *				95
PLA_30_HM200 *				95

* Blends extruded with a semi-industrial COMAC twin-screw extruder (up-scaled).

**Table 3:** Mechanical and MFR results of lab-scaled composites with different amounts of H0210 and HM200 HSP.

Blend Name	Elastic Modulus (GPa)	Stress at Break (MPa)	Elongation at Break (%)	Charpy Impact Resistance (C.I.S.) (kJ/m^2^)	MFR (g/10 min)
PLA	3.56 ± 0.21	58.94 ± 1.16	2.30 ± 0.33	2.53 ± 0.29	3.80 ± 0.51
PLA_20_H0210	4.03 ± 0.15	40.85 ± 0.76	1.35 ± 0.15	2.60 ± 0.32	22.69 ± 1.76
PLA_30_H0210	4.16 ± 0.03	33.77 ± 3.16	0.95 ± 0.24	2.73 ± 1.22	13.78 ± 1.98
PLA_40_H0210	4.26 ± 0.12	27.38 ± 0.97	0.89 ± 0.10	2.94 ± 0.61	8.49 ± 1.82
PLA_20_HM200	3.88 ± 0.12	30.34 ± 1.85	1.11 ± 0.24	2.45 ± 0.20	32.91 ± 3.17
PLA_30_HM200	4.13 ± 0.30	26.85 ± 2.19	1.10 ± 0.11	1.74 ± 0.24	34.08 ± 3.26
PLA_40_HM200	4.44 ± 0.20	16.80 ± 4.60	0.45 ± 0.17	1.73 ± 0.26	33.41 ± 2.93

**Table 4:** DSC first heating results for H0210 and HM200 PLA-based composites.

Blend Name	T_g_ (°C)	T_cc_ (°C)	T_m_ (°C)	Δ*H_cc_* (J/g)	Δ*H_m_* (J/g)	*X_cc_* (%)
PLA	61.8	105.7	172.2	32.4	44.9	13.5
PLA_20_H0210	58.2	94.3	170.9	21.8	32.4	14.2
PLA_30_H0210	57.2	93.3	169.3	22.4	33.3	16.8
PLA_40_H0210	57.2	94.2	168.8	18.9	27.8	16.0
PLA_20_HM200	55.2	91.0	168.0	26.8	38.2	15.2
PLA_30_HM200	54.3	88.6	167.1	23.1	35.2	18.6
PLA_40_HM200	53.7	87.4	166.8	20.9	33.0	21.7

**Table 5:** Mechanical and MFR results of the scaled-up HSPs composites.

Blend Name	Elastic Modulus (GPa)	Stress at Break (MPa)	Elongation at Break (%)	Charpy Impact Resistance (C.I.S.) (kJ/m^2^)	MFR (g/10 min)
PLA *	3.64 ± 0.19	64.60 ± 2.61	2.69 ± 0.14	2.51 ± 0.23	3.21 ± 0.55
PLA_30_H0210 *	4.30 ± 0.16	36.45 ± 1.00	1.09 ± 0.10	2.63 ± 0.35	6.23 ± 0.26
PLA_30_HM200 *	4.45 ± 0.11	38.42 ± 0.68	1.39 ± 0.18	2.29 ± 0.29	4.00 ± 0.59

* Blends extruded with a semi-industrial COMAC twin-screw extruder (up-scaled).

**Table 6:** DSC first heating results of scaled-up HSPs composites.

Blend Name	T_g_ (°C)	T_cc_ (°C)	T_m_ (°C)	Δ*H_cc_* (J/g)	Δ*H_m_* (J/g)	*X_cc_* (%)
PLA *	63.4	101.7	174.6	29.2	38.8	10.3
PLA_30_H0210 *	57.5	94.5	172.5	17.1	29.0	18.2
PLA_30_HM200 *	55.7	93.8	169.9	18.8	27.6	13.5

* Blends extruded with a semi-industrial COMAC twin-screw extruder (up-scaled).

## Data Availability

The data presented in this study are available on request from the corresponding author.
